# A double-layered liquid metal-based electrochemical sensing system on fabric as a wearable detector for glucose in sweat

**DOI:** 10.1038/s41378-022-00365-3

**Published:** 2022-05-07

**Authors:** Xuanqi Chen, Hao Wan, Rui Guo, Xinpeng Wang, Yang Wang, Caicai Jiao, Kang Sun, Liang Hu

**Affiliations:** 1 School of Biological Science and Medical Engineering, Beijing Advanced Innovation Center for Biomedical Engineering, Beihang University, Beijing, 100191 China; 2 Biosensor National Special Laboratory, Key Laboratory for Biomedical Engineering of Ministry of Education, Department of Biomedical Engineering, Zhejiang University, Hangzhou, 310027 China; 3 Department of Biomedical Engineering, School of Medicine, Tsinghua University, Beijing, China

**Keywords:** Electrical and electronic engineering, Materials science

## Abstract

Integrated electrochemical sensing platforms in wearable devices have great prospects in biomedical applications. However, traditional electrochemical platforms are generally fabricated on airtight printed circuit boards, which lack sufficient flexibility, air permeability, and conformability. Liquid metals at room temperature with excellent mobility and electrical conductivity show high promise in flexible electronics. This paper presents a miniaturized liquid metal-based flexible electrochemical detection system on fabric, which is intrinsically flexible, air-permeable, and conformable to the body. Taking advantage of the excellent fluidity and electrical connectivity of liquid metal, a double-layer circuit is fabricated that significantly miniaturizes the size of the whole system. The linear response, time stability, and repeatability of this system are verified by resistance, stability, image characterization, and potassium ferricyanide tests. Finally, glucose in sweat can be detected at the millimolar level using this sensing system, which demonstrates its great potential for wearable and portable detection in biomedical fields, such as health monitoring and point-of-care testing.

## Introduction

The detection of biological substances is of great significance in providing physiological information and indicating the state of life activities; thus, detection techniques have been widely utilized for medical diagnosis, health monitoring, and disease treatment. Among them, the electrochemical detection method is a widely studied and applied trace detection method that features high sensitivity and good specificity. Biosensors are the core element used to obtain important physiological information from living organisms, by which abundant physiological information can be collected through electrochemical sensing^1^. Increasing attention has been given to wearable electrochemical biosensing techniques due to the enormous demand for physiological health monitoring, the treatment of certain chronic diseases (such as diabetes and hypertension), and the diagnosis of communities and even families^2^. For example, a fully integrated closed-loop system based on a minimally invasive microneedle platform and wearable electronic device is studied. Combined with a flexible circuit board capable of signal analysis, processing, and transmission functions, the system can accurately monitor glucose fluctuations and effectively deliver insulin to regulate hyperglycemia in diabetic rat models^3^. Similarly, a fully integrated battery-free, wireless and flexible smart wound dressing is developed. On the one hand, a disposable stretchable electrode array based on a polyimide (PI) substrate can monitor changes in temperature, pH, and uric acid concentration to comprehensively report wound conditions. On the other hand, through the voltage control of the drug delivery module, the antibacterial cefazolin can be released for treating the infection with low power consumption^4^. In addition, an electrochemical biosensing system is reported, which combines mass-produced all-laser engraved multimodal sensors and flexible skin lab patches (flexible printed circuit modules). The study shows that it can continuously detect temperature, breathing rate, and low concentrations of uric acid and tyrosine during simultaneous sweat sampling, chemical sensing, and vital sign monitoring^5^. Furthermore, a wearable sensing platform that can be mass-produced and does not require batteries is proposed, and a free-standing friction nanogenerator (FTENG) based on a flexible printed circuit board (FPCB) is used to collect energy from human movement. The integrated and efficient power management system can power the multiplexed sweat biosensor and wirelessly transmit data to the user interface via Bluetooth during human testing^5^. Among these, the miniaturization and integration of the detection system with flexibility and conformability are of great value to achieve electrochemical detection and analysis.

At present, research on flexible wearable electrochemical sensing systems has made some progress. Studies on wearable biosensors have been reported with portable or even wearable detection systems on flexible substrates such as SEBS^6^, PI^7^, PDMS^8,9^, and PTFE^10^. Although they have excellent bending and stretching capabilities, poor air permeability will cause a certain burden on the skin when worn, potentially even causing skin inflammation. Therefore, the comfortability of wearable devices is greatly restricted^11–13^.

Fabric is a common skin-friendly material. A biosensor prepared by printing on fabric can be in direct contact with skin while also having increased air permeability and comfort. Studies on existing fabric-based biosensors have been carried out to detect biological signals such as glucose, pH, lactic acid, and uric acid^4,5,14^. These studies have applied and verified the functions of biosensors by implementing conductive circuits on fabrics. However, it is still challenging to fabricate complex circuits directly on fabric using common electrical inks, such as nano-copper^15^, nano-silver^16^, nano-gold^17^, and graphite ink^18^ for printing. This is mostly because the intersecting circuits require multilayer interconnection, which is difficult to achieve on fabrics with these conductive inks.

In this study, to overcome these challenges, we aim to fabricate an electrochemical sensing system on fabric using Galinstan (68.5% Ga, 21.5% In, and 10% Sn) as conductor, which is a liquid metal at room temperature. With great mobility and electrical conductivity, it has become an important candidate as a flexible conductor in the field of flexible electronics^1^. Moreover, the excellent mobility allows for self-healing and extensible properties, which are quite desirable in flexible and stretchable electronics^6,19^. Thus, in the present study, we achieve this electrochemical sensing system by printing all the circuits with liquid metal on two-layered fabric. Generally, the method of liquid metal patterning is complicated. For example, microchannel injection requires vacuum suction equipment^20^, atomized spraying requires a spray gun^21,22^, and 3D printing requires a series of related printing equipment^23^. Some studies require pretreatment of the liquid metal materials to make them adhesive, thereby reducing their fluidity for the subsequent molding step^24^. In addition, doping will also affect the conductivity of liquid metals^25^. In this paper, the unmodified liquid metal is patterned on the fabric with the help of polymethacrylate (PMA)^26^, without the need for additional equipment and complicated preprocessing. The glue enhances the adhesion between the metal and fabric and improves the stability of the circuit, and the circuit of the entire fabric substrate has good air permeability. In addition, the manufacturing process is convenient, quick, and user-friendly. This fabric electrochemical sensing system based on liquid metal integrates the functions of signal acquisition and signal processing by developing a double-layer liquid metal-based circuit. The system consists of the following main parts: a flexible fabric detection circuit based on liquid metal, control software on a laptop, and a replaceable electrode. The system can realize a constant voltage circuit at a range of 0–3 V for electrochemical detection and transfer the obtained data to the software on the laptop. The sensing part uses commercially available electrodes. The function of this electrochemical sensing system is verified by potassium ferricyanide tests. Moreover, the detection of glucose in artificial sweat is achieved at the millimolar level, which demonstrates its high potential for use in wearable electrochemical sensing. More experimental procedures and results are discussed below.

## Results and discussion

### Structure and function of the fabric electrochemical sensor system

**Fig. 1:**
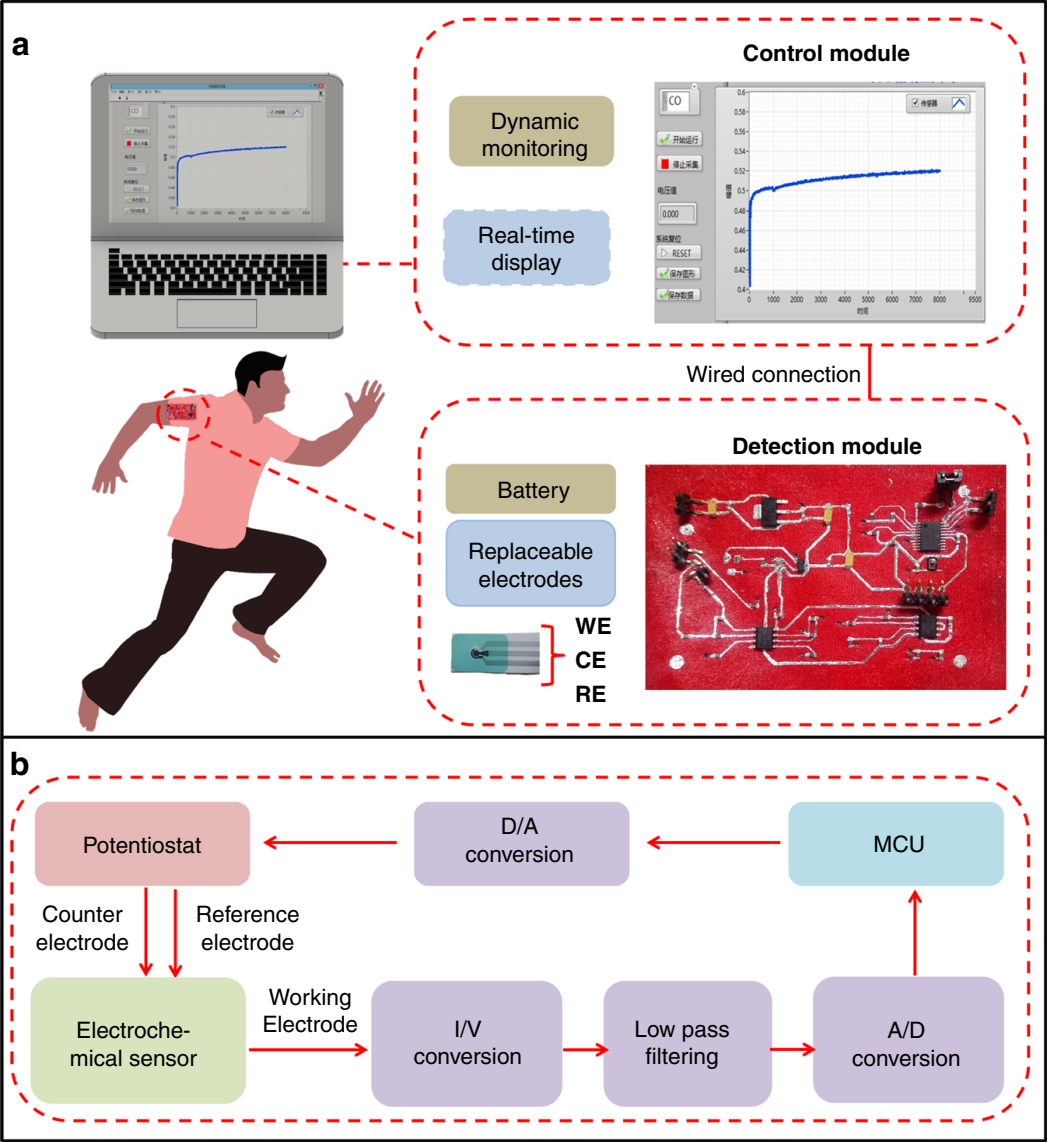
Schematic diagram and design of a fabric electrochemical sensor system based on liquid metal. **a** Electrochemical detection module of the whole fabric and the control module interface displayed on a laptop. **b** Working principle of the detection module.

The system consists of a flexible fabric circuit, a replaceable electrode for electrochemical detection, and software for circuit control and data analysis (Fig. 1a). The sensor system is based on the principle of electrochemical detection, and a weak signal detection circuit is designed to realize the collection, amplification, and transmission of current signals from nanoamps to microamps while eliminating interference signals. Notably, these signals can be displayed in real time on a laptop.

The detection module applies the electrodes at a constant potential and transmits the measured electrical signal to the software on the laptop (Fig. 1b). It contains several main modules: a power supply module, potentiostat module, I/V conversion module, amplification and filtering module, and digital-to-analog conversion module (Fig. S1 shows the circuit design of the detection module; Supplementary Material). The power module consists of a reference voltage core (REF3318) and a voltage regulator chip (SOT-233-AMS1117). The latter provides 3.3 and 1.8 V voltages for the potentiostat module. The voltage regulator module uses a precision amplifier (AD8572) to keep the voltage of the counter electrode (CE) and reference electrode (RE) consistent through a feedback mechanism. The I/V conversion and amplification filter modules amplify the weak response current through I/V conversion, eliminate interference signals through filtering, and theoretically can measure the microampere current. In the digital-to-analog conversion module, the processed electrical signal is sent to the microcontroller (STM32f042f4p6) ADC sampling terminal for processing, converted into a digital signal, and then input into software on a laptop.

Control software based on LabVIEW has been developed, which can realize the control and data collection of sensor detection on a laptop and display the sensor response curve in real time (Fig. 1a) (Fig. S2 shows the interface of the software on a laptop. On the left, different functions are provided, including serial port selection, start running, stop collection, real-time voltage value display, system reset, save graphics and save data operations; Supplementary Material).

**Fig. 2:**
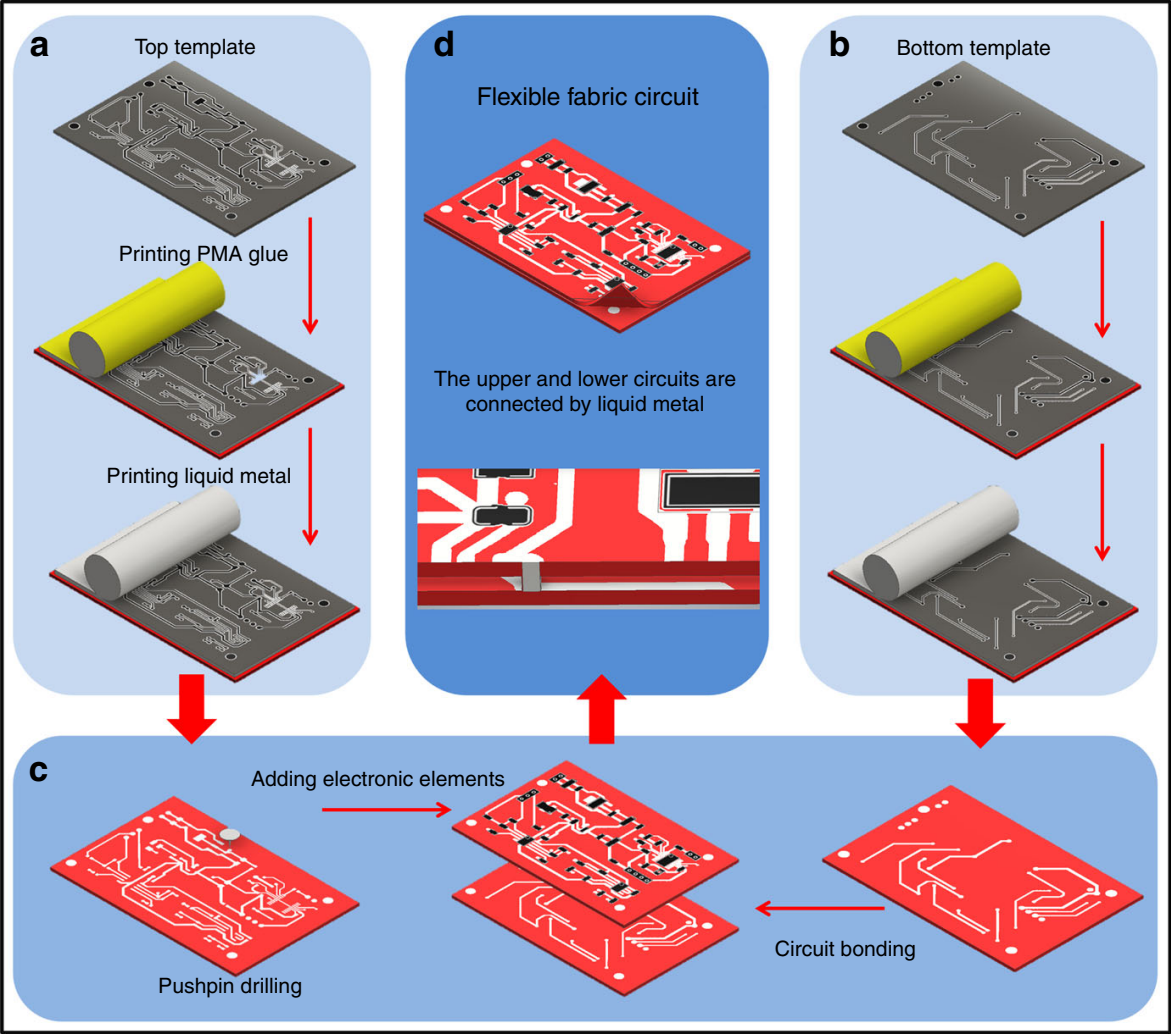
Manufacturing process of the flexible fabric circuit. The template method is used to print PMA glue and liquid metal on the fabric to form **a** upper circuit and **b** lower circuit. **c** The circuit intersection of the upper circuit is drilled, the electronic components are pasted, and then the two layers of circuits are overlapped. **d** Liquid metal is used to connect the upper and lower circuits to obtain a flexible fabric circuit.

The flexible fabric circuit of the detection module is based on 100% cotton fabric, unmodified liquid metal is used as the electric wire, and the template method is used for plane printing manufacturing (Fig. 2). The reason for using PMA glue is that gallium oxide on the surface of liquid metal can interact with PMA through hydrogen bonds, so the liquid metal can better adhere to the surface of the fabric^25^. Only applying glue to the area where the circuit wires are designed can ensure that the final product still retains the good air permeability of the fabric. The unmodified liquid metal still retains its good electrical conductivity^27^ without requiring complex pretreatment. Direct patterning of liquid metal is obviously simple and fast (Fig. S3b shows the finished picture of the flexible fabric circuit, Fig. S3c is the finished picture of the printed liquid metal wire of the upper circuit, and Fig. S3d is the finished picture of the liquid metal wire of the lower circuit; Supplementary Material).

The manufacturing of the detection circuit adopts a general template method and does not require additional processing of the manufacturing materials. The manufacturing method is simple, user-friendly, and fast, while retaining to the greatest extent the good air permeability of the fabric.

### Characterization of the flexible fabric circuit

**Fig. 3:**
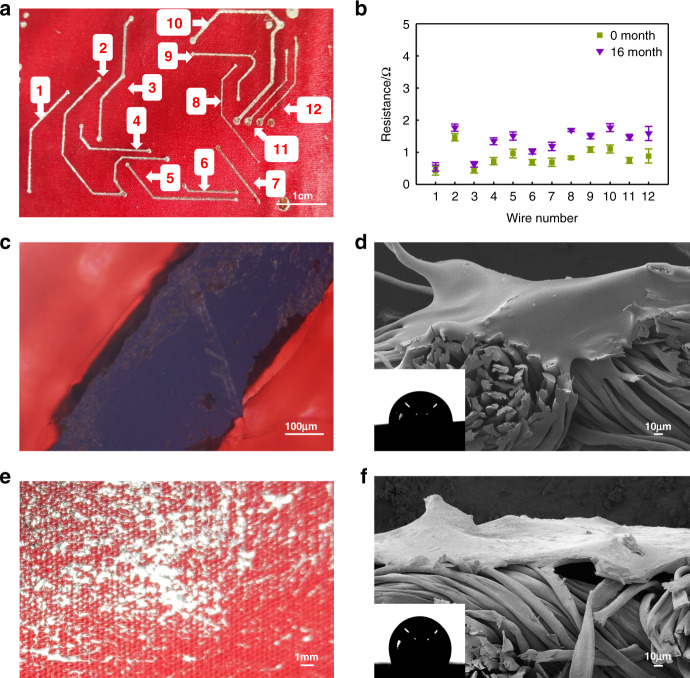
Electrical and physical characterization of the circuit. **a** Liquid metal wire on the fabric and its corresponding number. **b** Liquid metal wire resistance at Months 0 and 16. **c** Front view of the inverted microscope of the liquid metal wire. **d** Environmental scanning electron microscopy image of the liquid metal–glue–fabric cross-section. The inset is the contact angle measurement diagram of the corresponding interface. **e** Front view of liquid metal–fabric (no glue scraping). **f** Environmental scanning electron microscopy image of the liquid metal–fabric cross-section. The inset is the contact angle measurement diagram of the corresponding interface.

Figure 3a shows the liquid metal wires printed on the fabric and their corresponding numbers. Through measurement, we compare the line width corresponding to the liquid metal wire printed in the picture with the width of the design template (Table S1; Supplementary Material). Among the 12 wires, the maximum error between the printed width and the design width is 5.16% for Line 12, and the smallest error is 0.63% for Line 1. It can be found that the width of the printed wire is not much different from the design width. This shows that relying on the adhesion of PMA glue makes it possible to pattern the liquid metal on the fabric.

Next, we examine the resistance of the liquid metal wires in relation to the time variance (Fig. 3b). First, we observe the resistance of the printed liquid metal wire at Month 0. The difference in the length and width of the printed wire itself will lead to the difference in resistance. Line 2 is the longest, with a design length of 53.706 mm and a design line width of 0.508 mm. Its resistance is the largest among all lines, only 1.46 ± 0.10 Ω. Line 6 is the shortest, with a design length of 12.475 mm, a design line width of 0.508 mm, and a resistance of only 0.68 ± 0.07 Ω. The design line width of Line 8, Line 11, and Line 12 are only 0.381 mm, and the corresponding resistances are 0.82 ± 0.04 Ω, 0.74 ± 0.08 Ω, and 0.88 ± 0.19 Ω, respectively.

We compare the resistance of the printed liquid metal wire at Month 0 and Month 16, and it can be found that the resistance of each wire at Month 16 increases slightly. By comparison, it can be seen that the resistance of Line 6 is the largest (1.76 ± 0.12 Ω). In the circuit design, the minimum resistance is 33 Ω, except for R3, and the remaining resistances in the circuit are all kiloohms (for details, see S1; Supplementary Material). Therefore, compared with the resistance in the circuit, the resistance of the printed liquid metal wire is not large. The resistance of Line 6 is only 5.3% of the design circuit resistance R3. Even after being placed for a period of time, the resistance change of the liquid metal wire will not have a significant impact on the resistance of the entire circuit. This shows that the liquid metal wire on the fabric has certain time stability and can be used as the hardware basis of a reusable sensor system. The reason for the increase in electrical resistance may be due to the increased oxidation or corrosion in the air for a long time through SEM observation of the fabric–liquid metal part after 16 months. Therefore, the conductivity decreases (Fig. S4 suggests that after 16 months of storage, the oxide film on the surface of the liquid metal is rough; Supplementary Material).

In addition, we compared the contact resistance of electronic components and liquid metal wires at Months 0 and 6. Due to the excessive number of components, we randomly selected the contact resistances corresponding to the different pins of ten components (the results are shown in Fig. S6; Supplementary Material). At Month 0, when the circuit has just been prepared and connected, the contact resistances of the different electronic components and liquid metal wires are very small, between 0.1 and 0.2 Ω, and after 6 months, the contact resistances of the different components have increased. In the measured data, the maximum contact resistance value is measured on the first pin of the chip U1. However, even if the contact resistance increases, the measured maximum value does not exceed 2 Ω, indicating that the change in the contact resistance between the electronic component and the liquid metal wire has little effect on the circuit resistance.

By observing the liquid metal circuit under an inverted microscope (Fig. 3c), we can see that the liquid metal pattern is relatively straight and the edges are neat, indicating that the printed circuit is of good quality. The coverage area of the PMA glue is slightly wider than that of the liquid metal, and there is no glue covering on the surface of the fabric except for the contour of the liquid metal, which can retain the good air permeability of the fabric. Observing the cross-section of the fabric where the liquid metal is located (Fig. 3d), the liquid metal tightly covers the upper fiber of the fabric. Since liquid metal cannot form a specific pattern on the fabric without glue (Fig. 3e), the liquid metal is directly scraped on the fabric for subsequent research. By observing the cross-section of the liquid metal–fabric (Fig. 3f) and comparing the liquid metal–glue–fabric, we can see that there are still gaps between the liquid metal and fabric fibers in the cross-section of the liquid metal–fabric, and the liquid metal only thinly covers the surface of the fabric. The illustrations in Fig. 3d, f show the comparison of the contact angle of liquid metal on fabrics with and without glue, respectively. When on the glue-coated surface, the contact angle of the liquid metal drop is 85°. Directly on the cotton fabric, the contact angle of the liquid metal drop is 105°. In fact, the adhesion force has been measured in previous work, showing that the adhesion between PMA glue and liquid metal is ~2 mN, much larger than those between liquid metal and paper/fabrics (Fig. S8 shows the adhesion force between a liquid metal with PMA glue, paper, and fabric; Supplementary Material). From the above comparison, it can be seen that the liquid metal has better wettability on PMA glue than on the fabric. Thus, the combination with PMA glue realizes the patterning of the liquid metal on the fabric.

**Fig. 4:**
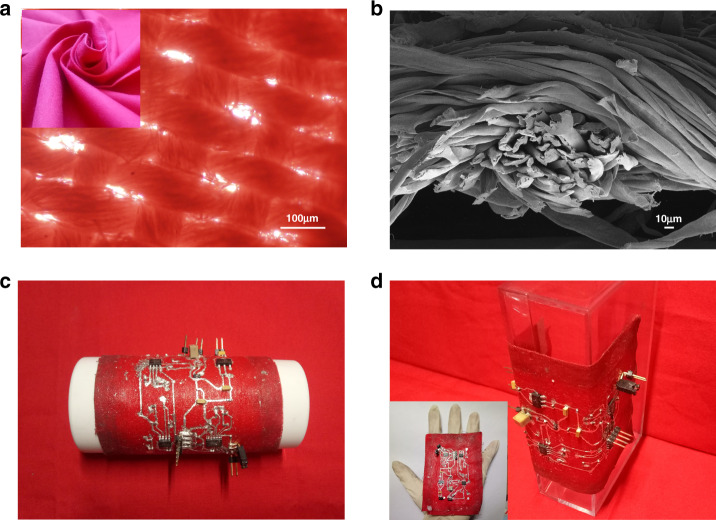
Characterization of the flexible fabric. **a** Inverted microscopy image of the 100% cotton fabric; the inset shows the flexibility of the 100% cotton fabric. **b** Environmental scanning electron microscopy image of 100% cotton fabric. **c** Curved flexible fabric circuit. **d** Bent flexible fabric circuit. The inset shows a flexible fabric circuit placed on the hand, with a length of 66 mm and a width of 40 mm. The thickness of the double-layer cotton fabric is only 0.4 mm.

Due to its own fiber characteristics and structural morphology, cotton fabric has good air permeability and water vapor resistance^28^. These properties are related to the hole diameter of the fabric, the number of warp and weft yarns used per centimeter, and the thickness of the fabric. The illustration in Fig. 4a shows the structure used in the flexible fabric circuit. The cotton fabric used is flexible and can be twisted at will. In the inverted microscope image in Fig. 4a, the gaps between the warp and weft yarns of the fabric can be observed. The scanning electron microscopy image in Fig. 4b shows a cross-section of a fabric woven by twisting multiple strands of yarn. The gaps between the multiple strands of yarns and the gaps left after warp and weft knitting provides the fabric with good air permeability. When making the circuit, only the liquid metal line pattern is connected with PMA glue, while the other parts of the entire flexible circuit are not glued. In this way, the breathability of the 100% cotton fabric can be retained to the greatest extent. Figure 4c, d shows the flexibility of the flexible fabric circuit. The thickness of the obtained double-layer fabric circuit is only 0.4 mm. The whole size of this flexible circuit is comparable to a palm with length and width dimensions of only 66 and 40 mm, respectively.

### Performance of the fabric electrochemical sensor system

The performance of the system is evaluated after the completed flexible fabric circuit is connected to a laptop. After the connection is successful, we select the correct serial port and baud rate in the software on the laptop. The software on the laptop successfully loads the control chip program. Then, the multimeter confirms that the power supply voltage, the operating voltage of the precision amplifier, and the voltage of the voltage regulator module are stable. Using the platinum disc electrode as the working electrode (WE), the voltage range^29^ is 0–0.6 V. Therefore, the potentiostatic voltage for potassium ferricyanide detection is set to 0.3 V. The purpose of potassium ferricyanide detection in this section is to study the performance of the sensor system itself, since a given constant potential voltage can catalyze the reduction of potassium ferricyanide and generate a reaction current. It is unnecessary to emphasize the selection of the potentiostatic voltage that maximizes the reaction current of potassium ferricyanide.

**Fig. 5:**
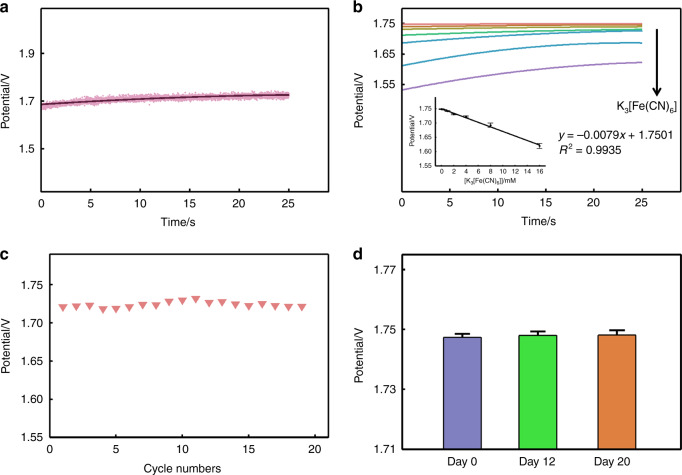
Performance of the fabric electrochemical sensor system. **a** Time–potential scatter plot and regression fitting curve of 4 mM potassium ferricyanide solution at a constant voltage of 0.3 V. **b** Time–potential regression fitting curves with 0, 0.5, 1, 2, 3, 4, 8, 16 mM potassium ferricyanide solutions at a constant voltage of 0.3 V, and the linear correlation between potential and concentration with a reaction time of 25 s. **c** Electric potential of a 4 mM potassium ferricyanide solution circulated nearly 20 times at a constant voltage of 0.3 V and a reaction time of 25 s. **d** Comparison of potentials of a 0 mM potassium ferricyanide solution at a constant voltage of 0.3 V on the 0th, 12th, and 20th days, and the reaction time is 25 s.

During the measurement of the electrochemical sensor system, the potentiostat module uses the RE potential as a reference to control the electrode potential of the WE and simultaneously detects the current flowing between the WE and CE for electrochemical detection. Because the current on the WE is too small, the current is converted into a voltage signal by the I/V module, and the interference signal is filtered out by the filter module. Its magnification is given by the following equation:

1 $$U = 2 \cdot R \cdot I$$

where *U* represents the voltage signal after amplification (V) and *I* represents the current signal before amplification (A). The amplification factor depends on the impedance used. In the design of the circuit, we use a resistor of *R* = 73.2 kΩ, which can effectively amplify the very small current. After the signal processing circuit, the voltage signal is sent to the ADC sampling terminal of the single-chip microcomputer. The sampling terminal can convert the analog signal into a digital signal. Therefore, the digital signal output by the single-chip microcomputer is transmitted to the laptop software, and the data are displayed in real time through software processing. Due to sampling and the sampling principle of the single-chip microcomputer, the data we obtain in real time are actually the display of the scatter diagram in Fig. 5.

As illustrated in Fig. 5, potassium ferricyanide solution is used to study the linearity, repeatability, and stability of the system. Figure 5a shows the time–potential scatter diagram of a 4 mM potassium ferricyanide solution at a constant voltage of 0.3 V. We find that although the scatter plot has a certain trend, there are also some marginal scatter points, which may be due to the removal of interference signals without filtering. To filter out the influence of interference on the scattered points, we fit the curve with a binomial based on the scatter plot, which is also very helpful for the subsequent discussion of the relationship between the concentration and output value. Figure 5b shows the time–potential fitting curve of potassium ferricyanide solution with a concentration of 0 mM to 16 mM. As the concentration is increased, the output potential decreases. The currents reach stability after 20 s. Thus, we choose the current at 25 s (*t* = 25) as the response of this sensor to the analyte. The amplified potential is linear and described by the following equation: *y* = (−0.0079 ± 0.0007) × *x* + (1.7510 ± 0.0050), *R*^2^ is 0.993. It can be seen that the designed system has a high correlation with the output in the concentration range of 0–16 mM, which demonstrates the effective current generated by the electrochemical reaction at a lower concentration (millimolar level). The system also has good linearity in processes, such as small current amplification, filtering, and digital-to-analog conversion.

Using the magnification relationship of Eq. (1), we can calculate the current responses to each concentration of potassium ferricyanide, as shown in Table 1. The current of 0.5 mM potassium ferricyanide is as low as 13.5 nA, and the current of 16 mM potassium ferricyanide is 0.875 μA. It can be found that the theoretical detection limit is also at the nanoampere level.

**Table 1:** Average current corresponding to each concentration of potassium ferricyanide in chronoamperometry, *t* = 25 s.

[Potassium Ferricyanide]/mM	0	0.5	1	2	4	8	16
Current/A	0	1.35 × 10^−8^	4.33 × 10^−8^	1.21 × 10^−7^	1.73 × 10^−7^	3.80 × 10^−7^	8.75 × 10^−7^

Next, the stability of the electrochemical sensing system itself is evaluated. Figure 5c displays the nearly 20 time–potential fitting curves with a 4 mM potassium ferricyanide solution. The corresponding potential outputs at *t* = 25 s are all at 1.72 V. The relatively stable potential output shows the repeatability of the electrochemical sensing system (Fig. S5a shows the corresponding time–potential fitting curve for nearly 20 times; Supplementary Material) Fig. 5d shows the corresponding potential values of the time–potential fitting curve at *t* = 25 s on the 1st day, 12th day, and 20th day with a 0 mM potassium ferricyanide solution (Fig. S5b shows the corresponding time–potential fitting curves for different days; Supplementary Material). Due to the need to discuss the impact of the number of days on the three sets of data, we use a single-factor analysis of variance to perform a significance analysis on the output potentials on the 1st and 12th days, as well as the 1st and 20th days. The *P* values are 0.40 and 0.45, respectively. The results show that the potential output on the 12th and 20th days is not significantly different from that on the 1st day, which shows the time stability of the system. This provides a reliable basis for the subsequent application detection of the system in a certain time span.

### Fabric electrochemical sensor system for glucose detection

**Fig. 6:**
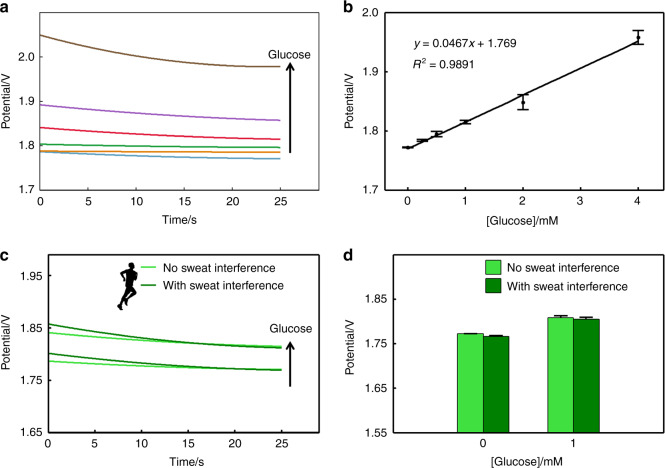
Fabric electrochemical sensor system for glucose detection. **a** Electrochemical sensor responses to glucose at concentrations of 0, 0.25, 0.5, 1, 2, 3, and 4 mM and a constant voltage of 0.55 V (time–potential regression fitting curve). **b** Linear relation between the sensor response and glucose concentrations with a 25 s reaction time. **c** Sensor responses to glucose (0 and 0.5 mM) with/without sweat interference at a constant voltage of 0.55 V. **d** Comparison of the potential between 0 and 1 mM glucose solutions with sweat interference, and the reaction time is 25 s.

Figure 6a shows the time–potential fitting curve of a glucose solution with a concentration of 0 mM to 4 mM. The scatter plot regression method still uses binomial regression. Figure 6b shows the linear correlation of the output, which can be described by the following equation: *y* = (−0.0467 ± 0.0068) × *x* + (1.7690 ± 0.0130), *R*^2^ is 0.9891. The designed system has a high correlation with the output in the concentration range of 0–4 mM. Similar to the potassium ferricyanide solution experiment, the system still maintains good linearity, but the concentration range of the glucose test is reduced. The lowest measurable concentration is 0.25 mmol/L. We speculate that the main reason is that the 1 mm nickel wire electrode is not modified, and the degree of glucose catalysis is limited. When the glucose concentration is too low, the reaction current is not obvious^30^.

Using the magnification relationship of Eq. (1), we can calculate the current corresponding to each concentration of glucose at *t* = 25 s, as shown in Table 2. The current of 0.25 mM glucose is as low as 80.1 nA, and the current of 4 mM glucose is 1.30 μA. Even after the electrode is replaced by an electrochemical reactant, the current is still in the nanoampere to microampere range. The signal collection shows that the overall electrochemical sensor system designed has strong applicability and relatively stable amplification.

**Table 2:** Average current corresponding to each concentration of glucose in chronoamperometry, *t* = 25 s.

[Glucose]/mM	0	0.25	0.5	1	2	4
Current/A	0	8.01 × 10^−8^	1.53 × 10^−7^	2.92 × 10^−7^	5.21 × 10^−7^	1.30 × 10^−6^

Next, the feasibility of testing the glucose concentration in sweat is verified by comparing the curve of glucose solution in sweat with that without sweat. The time–potential fitting curves of the 0 mM and 1 mM glucose solutions with and without sweat interference components shown in Fig. 6c are compared with the outputs in the histogram. The curves of the same concentration all have a higher degree of coincidence. The specific difference can be compared by observing the potential of the curve at *t* = 25 s, as shown in the histogram in Fig. 6d. In particular, the significant difference between the presence and absence of sweat interference components in 1 mM glucose solution is calculated. As the interference component is a single factor, we use a single factor analysis of variance to obtain a *P* value of 0.0647. The results show that there is no significant difference in whether the 1 mM glucose solution with the interference component of sweat is added. Therefore, when the system is used to detect glucose in sweat, the main components in sweat (sodium chloride, ammonium chloride, urea, lactic acid, and acetic acid) basically do not affect glucose detection. Thus, the feasibility of using nickel as a WE for the selective detection of glucose in sweat is verified.

It can be found that the fabric electrochemical sensing system based on liquid metal is a reusable platform with replaceable electrodes. The results verify and execute the complex functions of the flexible circuit on the fabric through a simple printing method while miniaturizing and integrating the sensing and analysis modules, thereby demonstrating its great potential for long-term wearable electrochemical monitoring. We measured the performance of this electrode under bending and stress effects. However, the signals clearly drift, which interferes with the sensor response in some cases. This result should be due to the variation in the interconnect resistance and contact resistance during the bending process, which significantly increases the noise. However, the system can remain stable and executable when it is placed on a certain curved surface in a static state. This stability of the whole system remains to be improved for practical applications, on which we will make more efforts in the future. We have also discussed this in the revised manuscript.

In summary, a miniaturized fabric-based electrochemical sensing system is introduced by fabricating a two-layered circuit using liquid metal, and electrochemical sensing is realized. Owing to the fluidity of liquid metal and the air permeability of fabric, this compact electrochemical system renders great comfort and portability for wearable sensing devices. It can realize a constant voltage circuit in the range of 0–3 V, which can be used for concentration detection. The performance of this system is verified by a ferricyanide test. Moreover, glucose at millimolar levels has been detected with the interference of artificial sweat, which further proves its sensitivity and stability. Above all, this wearable and compact electrochemical system on fabric show high potential applicability for health monitoring, home diagnosis and treatment, mobile medicine, and point-of-care testing.

## Materials and methods

### Materials

The fabric was observed with an inverted microscope (OLYMPUS IX71, Japan) and an environmental scanning electron microscope (ZEISS-Ultra55, Germany)

The adhesion force was measured with a standard contact angle measuring instrument (SL200B, American).

Liquid metal (Galinstan consisting of 68.5 wt% gallium, 21.5 wt% indium, and 10 wt% tin), 100% cotton fabric, and artificial sweat were commercially purchased and used as received.

A 1 mm nickel wire electrode, 3 mm platinum disc electrode, 15*15*0.1 mm platinum sheet electrode, Ag/AgCl electrode, and Hg-HgO RE were purchased from Shanghai Yueci Electronic Technology Co., Ltd., Shanghai, China.

Glucose, potassium ferricyanide, and potassium chloride were purchased from Sinopharm Chemical Reagent Co., Ltd., Shanghai, China.

Electronic components, such as resistors and capacitors, were purchased from Shenzhen Lichuang Oline Mall, Shenzhen, China.

### Fabrication of a liquid metal-based electrochemical system on fabric

The liquid metal-based electrochemical system is screen printed on a thin layer of cotton via the adhesion of PMA. First, the conductive path of the designed circuits (Fig. S3a) is patterned into two layers on stainless boards (80 mm × 55 mm × 1 mm) as screen printing templates by laser processing. Then, the template is placed on the cotton, and a layer of PMA glue is printed. Using the same printing method, a layer of Galinstan is coated on the PMA patterns to form conductive lines. Electronic components are glued into the circuit and connected to liquid metal wires, such as chip resistors, capacitors, and chips. Next, the upper and lower layers of the circuit are overlapped based on the four positioning points of the circuit. To connect the two layers, the interconnecting open points are overlaid, and additional liquid metal is added to connect the two layers of circuits. The multimeter is used to check the resistance to confirm the continuity at the intersection of the upper and lower circuits, and finally, a flexible fabric circuit is obtained.

### Characterization of the flexible fabric circuit

We conducted a conduction test, stability test, and morphological characterization of the liquid metal wire on the fabric.

#### Conduction test

First, a multimeter (VICTOR: VC890C, China) was used to test the resistance of different lengths of liquid metal wires that were not connected to the electronic components. The size of the resistance indicated whether the printed liquid metal circuit was connected.

#### Stability test

The printed fabric was exposed to air for 16 months. The multimeter measured the resistance of the liquid metal wire again and compared it with the initial resistance to determine whether the liquid metal wire could still be turned on after a certain period of time. This could simply confirm the stability of the liquid metal pattern and was also the basis for the stability of the entire fabric electrochemical system.

#### Morphological characterization

An inverted microscope and a field-emission environment scanning electron microscope were used to observe the liquid metal pattern on the fabric to study its microstructure morphology. Through the resistance test and microcharacterization of the liquid metal pattern, the feasibility of the liquid metal pattern as a complex function circuit line was studied from different angles, and the resistance test was carried out at different times to explore its sustainable utilization potential. In addition, through the field-emission environment scanning electron microscope cross-sectional observation and contact angle measurement of the liquid metal fabric connected with and without PMA glue, the role of PMA glue in the formation of liquid metal was discussed.

To characterize the characteristics of the substrate of the flexible fabric circuit, 100% cotton fabric under different angles was observed. We measured the size of the flexible fabric circuit. An inverted microscope was used to observe the front of the fabric, and an environmental scanning electron microscope was used to observe the cross-section of the fabric to study its microscopic morphology. We observed the hole diameter of the fabric, the number of warp and weft yarns, and the thickness of the fabric through photos to explore the breathability of the 100% cotton fabric.

### Electrochemical test of the system

After connecting the detected circuit, Keil uVision5 software (on a laptop) was used to download the program to the microcontroller (STM32f042f4p6) to realize the design function. After connecting the system, a multimeter was used to test whether the chip met the design working voltage and whether the three electrodes met the design constant potential.

A potassium ferricyanide/potassium chloride aqueous solution was selected as the test solution to test the performance of the entire fabric electrochemical sensor system. Using chronoampere detection, the tiny current generated by redox was displayed on the laptop in real time through the I/V conversion and data transmission of the sensor system. Due to the amplification of the detection circuit, the real-time curve displayed on the laptop was a time–potential curve. By testing and comparing the time–potential curves of different concentrations of potassium ferricyanide solutions, the linearity, repeatability, and stability of the electrochemical sensor system could be studied.

Seven sets of potassium ferricyanide/potassium chloride aqueous solutions were prepared, and the potassium ferricyanide concentrations were 0, 0.5, 1, 2, 4, 8, and 16 mM, while the potassium chloride concentration was 0.1 mM. Each experiment used 2 ml of potassium ferricyanide/potassium chloride aqueous solution. The constant voltage of the detection circuit was adjusted to 0.3 V. The platinum disc electrode, platinum sheet electrode, and Ag/AgCl electrode were used as the WE, CE, and RE, respectively. The time calculated by chronoamperometry was *t* = 25 s.

### Electrochemical detection of glucose in sweat

The system was tested with different concentrations of glucose solutions and the linear relationship of glucose detection was studied. By adding components representing artificial sweat interference to the glucose solution, the time curve of glucose with and without sweat was compared, and the feasibility of the system for detecting glucose in sweat was explored.

Six groups of glucose/sodium hydroxide aqueous solutions were prepared, and the glucose concentrations were 0, 0.25, 0.5, 1, 2, and 4 mM. The concentration of sodium hydroxide in each experiment was 0.02 mM, and the glucose/sodium hydroxide aqueous solution was 2 ml. In addition, two sets of glucose solutions with concentrations of 0 and 1 mM were prepared, a total of 10 ml was used, and contained artificial sweat interference components. The main interference components in the artificial sweat included sodium chloride, ammonium chloride, urea, lactic acid, and acetic acid. The constant voltage of the detection circuit was adjusted to 0.55 V. A 1 mm nickel wire electrode, platinum sheet electrode, and Hg-HgO RE were used as the WE, CE, and RE, respectively. The time calculated by chronoamperometry is *t* = 25 s.

## Supplementary information

Supplementary material
