# Supplementary material for: A double-layered liquid metal-based electrochemical sensing system on fabric as a wearable detector for glucose in sweat
Source: Microsyst Nanoeng. 2022 May 7;8:48. doi: 10.1038/s41378-022-00365-3 (PMC9079077; doi:10.1038/s41378-022-00365-3)
Supplement: Supplementary file 1 — Supplementary material [file 41378_2022_365_MOESM1_ESM.docx]

##### Supplementary material

**A double-layered liquid metal-based electrochemical sensing system on fabric as a wearable detector for glucose in sweat**

Xuanqi Chen^1#^, Hao Wan^2#^, Rui Guo ^3^, Xinpeng Wang^1^, Yang Wang^1^, Caicai Jiao^1^, Kang Sun^1^, Liang Hu^1*^

1. Beijing Advanced Innovation Center for Biomedical Engineering, Beihang University, Beijing 100191, China
2. Biosensor National Special Laboratory, Key Laboratory for Biomedical Engineering of Ministry of Education, Department of Biomedical Engineering, Zhejiang University, Hangzhou, 310027, China

Department of Biomedical Engineering, School of Medicine, Tsinghua University, Beijing, China# These authors contributed equally to this work

* Corresponding author: cnhuliang@buaa.edu.cn

**Table S1 Comparison of design line width and printed line width of liquid metal wire**

| Wire Number | Design line width /mm | Printed line width /mm | Erorr |
| --- | --- | --- | --- |
| 1 | 0.508 | 0.504±0.008 | 0.63% |
| 2 | 0.508 | 0.513±0.007 | 1.01% |
| 3 | 0.635 | 0.646±0.001 | 1.88% |
| 4 | 0.508 | 0.523±0.018 | 2.98% |
| 5 | 0.508 | 0.515±0.007 | 1.43% |
| 6 | 0.508 | 0.496±0.019 | 2.27% |
| 7 | 0.508 | 0.514±0.013 | 1.30% |
| 8 | 0.381 | 0.383±0.018 | 0.74% |
| 9 | 0.508 | 0.521±0.020 | 2.56% |
| 10 | 0.762 | 0.798±0.019 | 4.81% |
| 11 | 0.381 | 0.377±0.028 | 0.89% |
| 12 | 0.381 | 0.400±0.021 | 5.16% |


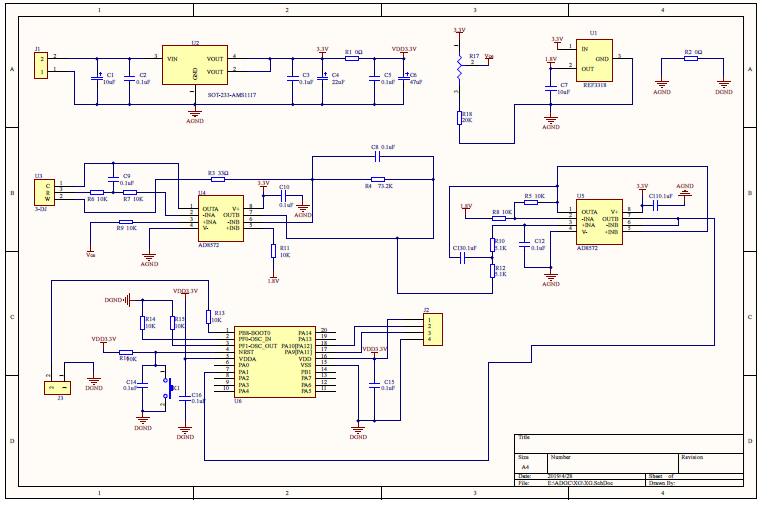


**Fig. S1 Design circuit diagram of flexible fabric circuit.**


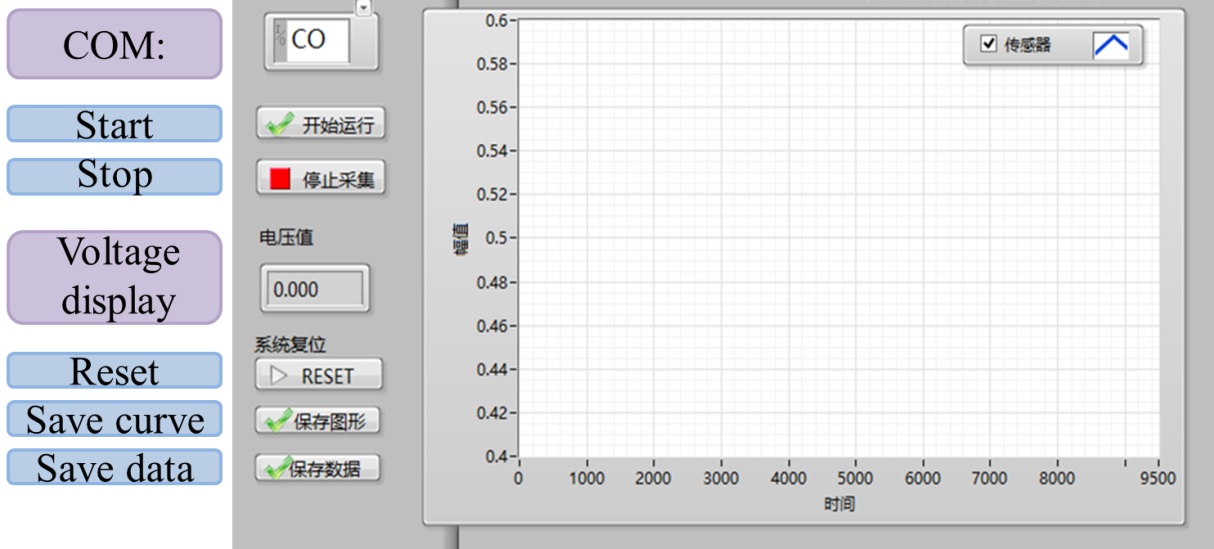


**Fig. S2** **The software on laptop.** The software on laptop display interface, which can control the start and stop of detection, display voltage value, real-time display curve, save curve, change abscissa of time and ordinate of response.


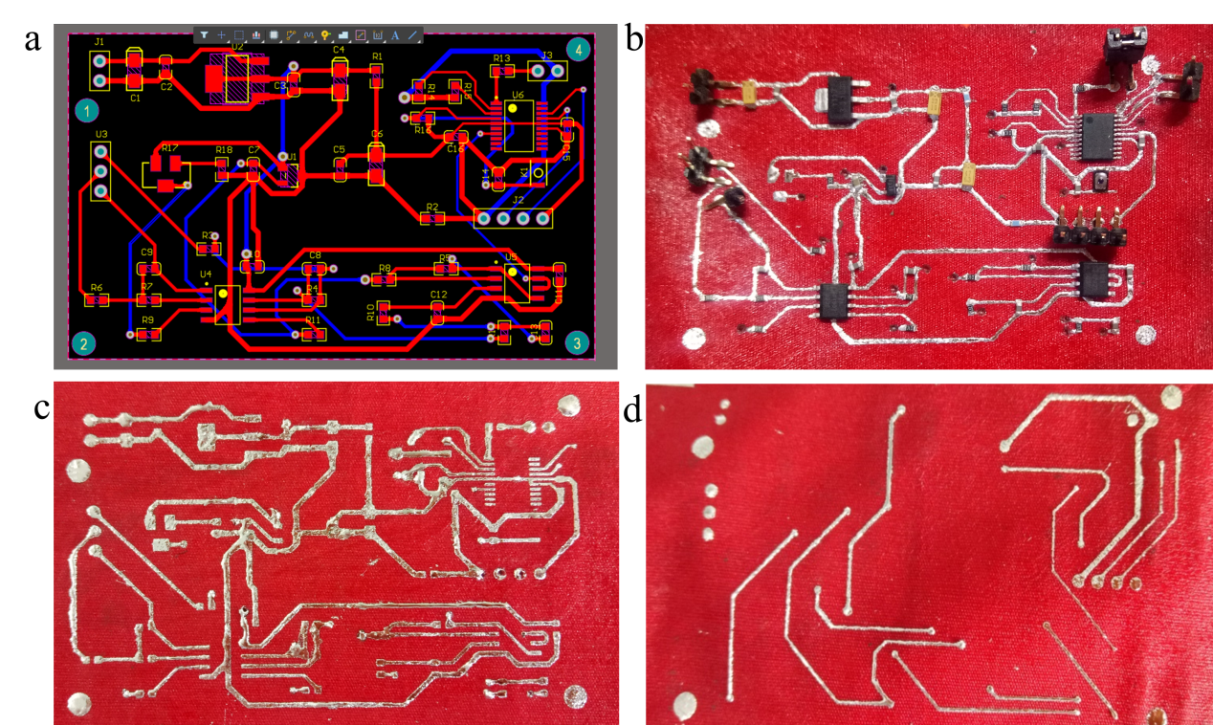


**Fig. S3 Flexible fabric circuit. a** PCB of flexible fabric circuit. **b** Photograph of flexible fabric circuit. **c** Printed picture of upper circuit of flexible fabric circuit. **d** Printed physical picture of the lower circuit of flexible fabric circuit.


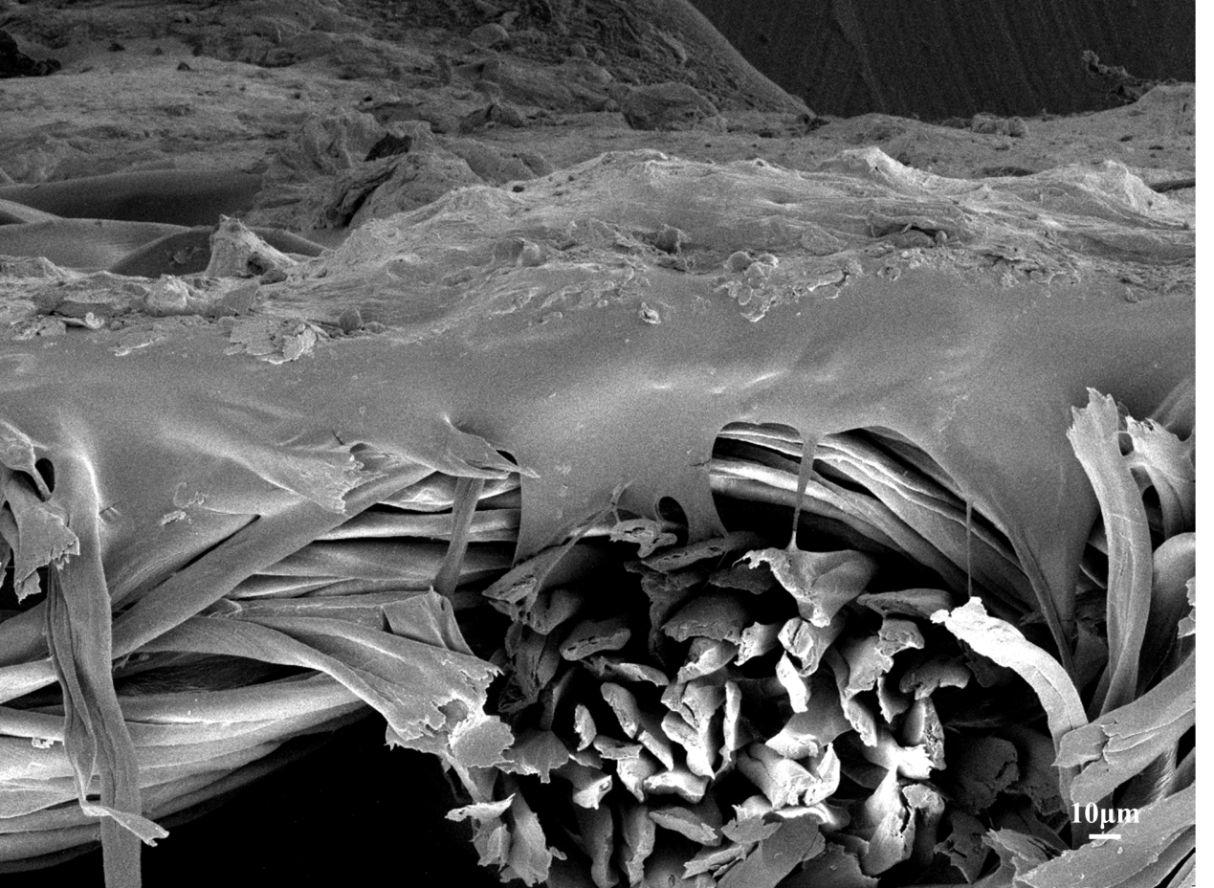


**Fig. S4 Characterization of circuit.** The environmental scanning electron microscope image of the field emission environment of the liquid metal-glue-fabric cross-section after being placed for 16 months.


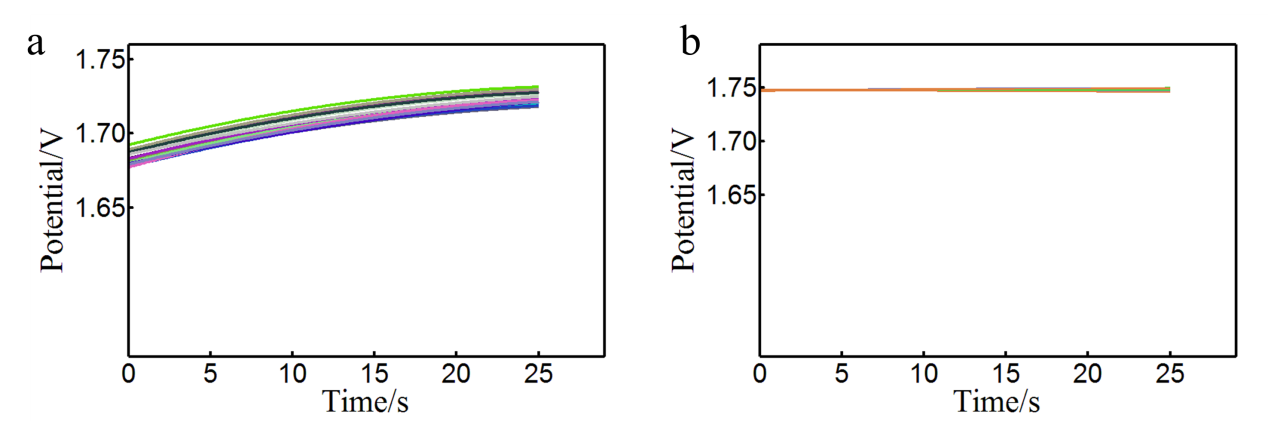


**Fig. S5 Performance of fabric electrochemical sensor system. a** Time-potential regression curve of 4 mM potassium ferricyanide solution at 0.3 V constant voltage for nearly 20 cycles, reaction time 25 s. **b** Time-potential regression line of 0 mM potassium ferricyanide solution at a constant voltage of 0.3 V on the 0th, 12th, and 20th days, the reaction time is 25 s.


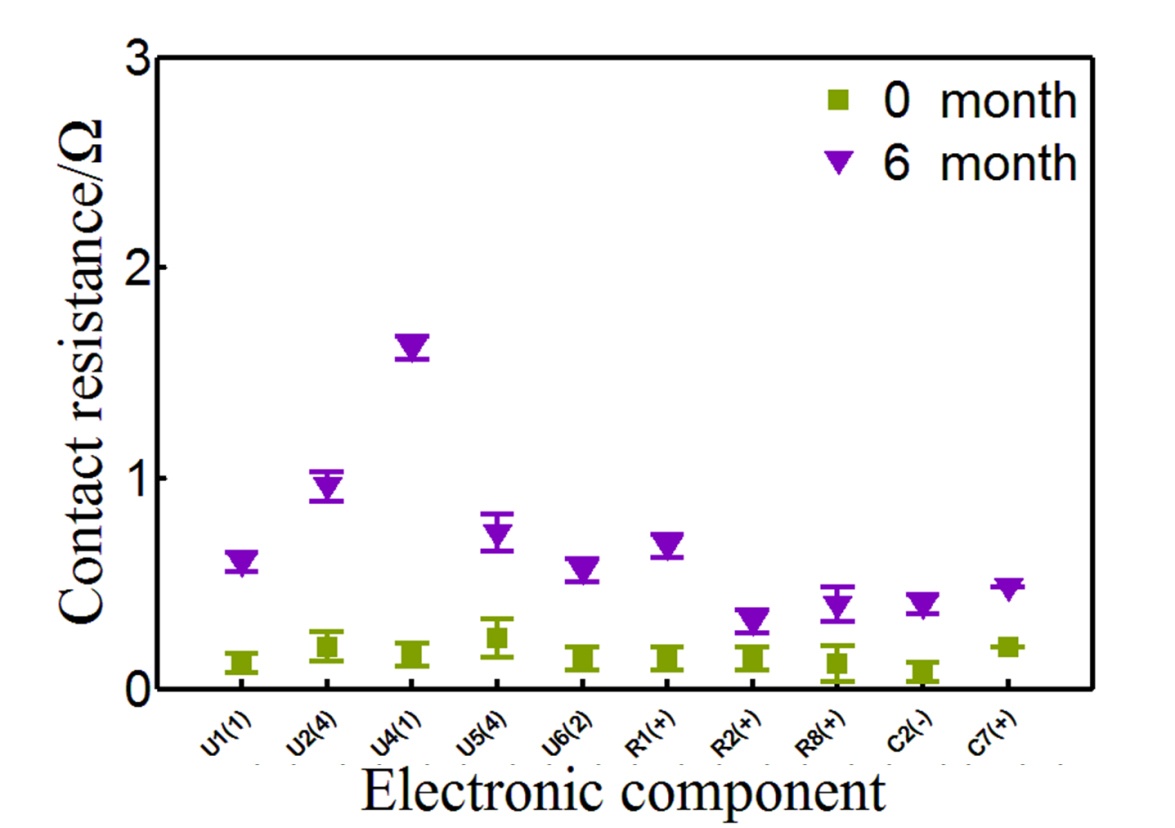


**Fig. S6 Measurement of contact resistance.** The results show the contact resistance at month 0 and 6 , the horizontal axis represents randomly selected electronic components, the numbers in brackets represent the corresponding pins of the chip, and the positive and negative signs indicate that the resistance or capacitance are connected to the circuit at a higher (+) or lower(-) potential, the vertical axis represents the corresponding contact resistance.


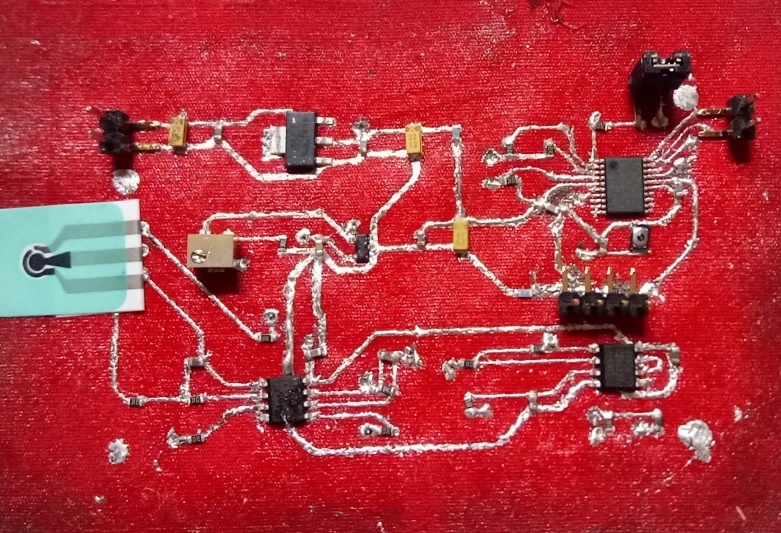


**Fig. S7 The electrochemical system with a screen printed electrode.**


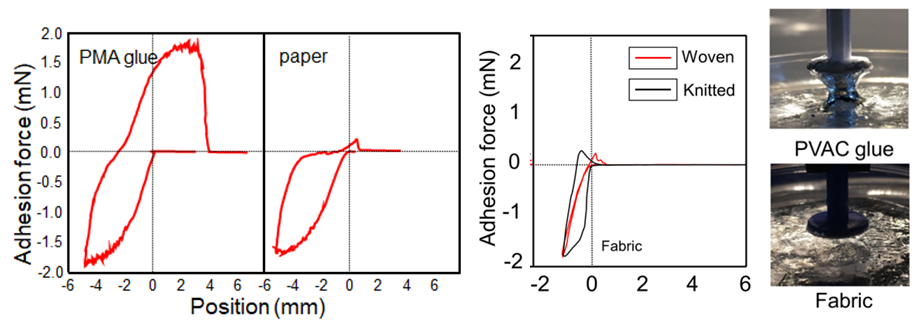


**Fig. S8 The adhesion force between liquid metal with PMA glue, paper**^1^ **and fabric**^2^**.**

**References**

1. Guo, R. et al. One‐Step Liquid Metal Transfer Printing: Toward Fabrication of Flexible Electronics on Wide Range of Substrates. *Adv. Mater. Technol*. **3**, 1800265 (2018).

2. Guo, R. et al. Semiliquid Metal Enabled Highly Conductive Wearable Electronics for Smart Fabrics. *ACS Appl. Mater. Inter.* **11**, 30019-30027 (2019).
